# Glymphatic surrogate, choroid plexus remodeling, and exploratory brain network analysis in the post-acute phase of anti-NMDAR encephalitis: multimodal MRI evidence for cognitive associations and differential white matter mediation

**DOI:** 10.3389/fimmu.2026.1918658

**Published:** 2026-08-31

**Authors:** Run Du, Jinyu Tao, Junwei Xiong, Yayun Xiang, Qiyuan Zhu, Zhuowei Shi, Wenwen Wang, Yongmei Li, Chun Zeng

**Affiliations:** 1 Department of Radiology, the First Affiliated Hospital of Chongqing Medical University, Chongqing, China; 2 Department of Otorhinolaryngology, Banan Hospital of Chongqing Medical University, Chongqing, China

**Keywords:** ALPS index, anti-N-methyl-D-aspartate receptor encephalitis, brain network, choroid plexus, cognitive function, glymphatic system, multimodal MRI, tract-based spatial statistics

## Abstract

**Background:**

Anti-N-methyl-D-aspartate receptor (anti-NMDAR) encephalitis typically presents with acute neuroinflammation, and many patients often experience persistent cognitive impairment during the post-acute/recovery phase. While impaired cerebrospinal fluid (CSF)-related markers—including glymphatic function and choroid plexus (CP) structural remodeling—are well documented in neurodegenerative diseases, their role in acute autoimmune brain injury remains unclear.

**Objective:**

To evaluate a glymphatic surrogate (diffusion tensor image analysis along the perivascular space (DTI-ALPS) index), choroid plexus structural remodeling (CP/intracranial volume (ICV) ratio), white matter microstructural integrity (global fractional anisotropy (FA) using tract-based spatial statistics (TBSS)), and structural covariance network (SCN) topology in anti-NMDAR encephalitis using multimodal MRI, and to investigate their associations with cognition, with an exploratory mediation analysis examining whether white matter microstructure mediates these associations.

**Methods:**

A total of 103 anti-NMDAR encephalitis patients and 97 healthy controls (HCs) with comparable sex distribution were enrolled. The glymphatic surrogate was evaluated by DTI-ALPS and CP structural remodeling was quantified by CP. White matter microstructural differences were analyzed using TBSS; mean FA values were extracted from all statistically significant clusters within the white matter skeleton (FA threshold = 0.2) after TFCE correction (*P* < 0.05), and the mean FA across these clusters was used as a global measure. Group-level SCNs were constructed based on 68 cortical regions, and graph theory metrics were compared. Partial correlation and bootstrap mediation analysis (5, 000 resamples) examined relationships among CSF markers, FA, and cognitive scores (MoCA, MMSE). A Monte Carlo sensitivity analysis was performed to determine the minimum indirect effect detectable at the achieved sample size (n = 103).

**Results:**

Compared to HCs, patients with anti-NMDAR encephalitis showed significantly decreased bilateral DTI-ALPS indices (left: 1.361 ± 0.196 vs. 1.489 ± 0.178; right: 1.335 ± 0.183 vs. 1.483 ± 0.174; *q =* 0.0001), increased CP/ICV ratio (0.080 ± 0.026 vs. 0.071 ± 0.019, *q =* 0.0135), and reduced global FA (0.506 ± 0.052 vs. 0.597 ± 0.099, *q<* 0.0001). In covariate-adjusted analyses within the patient group (n=103), the CP/ICV ratio showed strong negative correlations with MoCA (partial *r* = −0.463, *q<* 0.001) and MMSE (partial *r =* −0.317, *q =* 0.0078), while the left DTI-ALPS index showed a significant positive correlation with MoCA (partial *r =* 0.310, *q =* 0.0078). Global FA was strongly correlated with the DTI-ALPS index (partial *r =* 0.394, *q<* 0.001). However, exploratory bootstrap mediation analysis revealed that global FA significantly mediated the DTI-ALPS-MoCA association (indirect effect = 0.075, 95% CI [0.011, 0.152]; proportion mediated = 70.57%), but did not mediate the CP/ICV-cognition associations (all 95% CIs included zero). Sensitivity analysis indicated adequate power to detect indirect effects ≥ 0.13; the observed effect (0.075) fell below this threshold, warranting caution in interpretation. Exploratory group-level structural covariance network analysis revealed topological changes in patients, including increased clustering coefficient and modularity and decreased global efficiency; however, these network metrics could not be linked to individual cognitive or CSF measures due to the group-level nature of network construction.

**Conclusions:**

Patients with anti-NMDAR encephalitis exhibit concurrent glymphatic function, CP structural remodeling, widespread white matter microstructural damage, and topological reorganization. The CP/ICV ratio emerged as the primary imaging correlate of cognitive dysfunction. Although exploratory mediation suggested that FA partially accounted for the ALPS-cognition association, this likely reflects shared methodological collinearity rather than a distinct biological pathway, reinforcing CP/ICV as the more robust clinically relevant marker.These findings extend the concept of glymphatic-choroid plexus pathology from neurodegenerative diseases to post-acute autoimmune brain injury, providing a framework for non-invasive biomarkers in anti-NMDAR encephalitis.

## 1 Introduction

Anti-N-methyl-D-aspartate receptor (anti-NMDAR) encephalitis is a severe autoimmune disorder of the central nervous system (CNS) mediated by autoantibodies (1, 2). It typically causes acute neuroinflammation, blood-brain barrier (BBB) disruption, and long-term cognitive impairment (3, 4), with many patients entering a prolonged recovery phase during which persistent structural and functional abnormalities may be observed. Although immunotherapy has improved acute outcomes, many patients still suffer from persistent cognitive deficits (4, 5). The mechanisms of structural and functional brain damage remain incompletely understood.

Cerebrospinal fluid (CSF)-related markers, including glymphatic function and choroid plexus (CP) structural remodeling, constitute a key homeostatic mechanism for waste removal, fluid balance, and immune regulation (6, 7). Importantly, the diffusion tensor image analysis along the perivascular space (DTI-ALPS) index), is a non-invasive surrogate of glymphatic diffusivity derived from diffusion tensor imaging, while CP/ICV reflects inflammation-related structural remodeling rather than a direct measure of fluid flow; these two markers index related but distinct physiological processes. The glymphatic system facilitates convective influx of CSF along perivascular spaces and efflux of interstitial solutes; this process depends critically on aquaporin-4 (AQP4) channels on astrocytic endfeet and is highly sensitive to neuroinflammation and tissue damage (5, 8, 9). Anti-NMDAR antibodies can disrupt AQP4 polarity on astrocytic endfeet, induce perivascular space obstruction, and trigger choroid plexus (CP) hypertrophy as an immune gateway, thereby impairing CSF circulation and glymphatic function. The CP, as the main source of CSF and a gateway for immune cell entry, undergoes structural and functional remodeling during brain inflammation (10, 11). Its volumetric changes directly reflect the neuroinflammatory burden and have increasingly been recognized as an imaging biomarker (12, 13). Accumulating evidence links CSF-related marker impairment to neurodegenerative diseases such as Alzheimer’s disease and Parkinson’s disease, where these markers are often associated with white matter injury (14, 15). However, whether and how CSF-related markers are disrupted in acute autoimmune brain injury, and whether white matter damage mediates the relationship between such dysregulation and cognitive impairment in anti-NMDAR encephalitis, remain largely unknown.

Beyond CSF homeostasis, neuroimaging studies have confirmed that anti-NMDAR encephalitis triggers prominent topological reorganization of brain structural networks. This reorganization is characterized by impaired network integration, abnormal hub distribution, and compensatory changes in local connectivity, particularly affecting cognition-related networks such as the default mode network and the limbic system (16–18). In parallel, tract-based spatial statistics (TBSS) applied to diffusion tensor imaging (DTI) have revealed extensive white matter microstructural injury in anti-NMDAR encephalitis, manifesting as reduced fractional anisotropy (FA) (19, 20). These findings indicate that disrupted white matter tract integrity is a core feature of brain injury in this condition. Nevertheless, the interactions among CSF-related markers, CP volume abnormalities, white matter microstructural damage, and brain network reorganization—and the specific pathways through which they mediate cognitive impairment—have not been systematically explored in anti-NMDAR encephalitis.

Clarifying these interactions is critical for identifying novel imaging biomarkers and therapeutic targets. Multimodal magnetic resonance imaging (MRI) provides a feasible tool to dissect these pathological processes. The DTI analysis along the perivascular space (DTI-ALPS) index quantitatively evaluates diffusion efficiency along perivascular spaces and serves as a non-invasive glymphatic surrogate, although it is confounded by white matter microstructure as cautioned by Taoka (21). Normalized CP volume quantifies inflammation-related structural alterations (22). TBSS enables detection of whole-brain white matter microstructural injury. structural covariance network (SCN) comprehensively characterize the topological reorganization of brain networks (23, 24).

To address these gaps, this study used multimodal MRI to systematically characterize glymphatic surrogate (DTI-ALPS), CP structural remodeling, whole-brain white matter microstructure, and topological features of brain structural networks in a large cohort of patients with anti-NMDAR encephalitis during the post-acute stage. We examined changes in the DTI-ALPS index and relative CP volume, assessed abnormal whole-brain FA patterns using TBSS, and analyzed topological changes in morphological covariance networks. We further explored the associations among these markers, white matter microstructural injury, brain network reorganization, and cognitive function. Specifically, we sought to clarify the distinct patterns of these pathological alterations and to determine, through exploratory mediation analysis, whether white matter microstructural damage mediates the association between CSF-related markers and cognitive impairment.

## 2 Materials and methods

### 2.1 Study population and ethical approval

Between July 2017 and July 2025, 132 consecutive patients with a clinical diagnosis of anti-NMDAR encephalitis were initially screened at the First Affiliated Hospital of Chongqing Medical University. Of these, 29 were excluded: 12 for missing or incomplete MRI data, 9 for severe motion artifacts on DTI sequences precluding quantitative analysis, 5 for coexisting CNS disorders (3 with prior stroke, 2 with brain tumors), and 3 for incomplete cognitive assessment data. The final patient cohort comprised 103 individuals. Additionally, 97 healthy controls (HCs) with comparable sex distribution were enrolled, alongside 103 patients with anti-NMDAR encephalitis.

The inclusion criteria for the patient group followed the diagnostic criteria proposed by Graus et al. (2016) (25), requiring positive detection of anti-NMDAR antibodies in either CSF or serum. Exclusion criteria for all participants included: (1) coexisting CNS disorders (e.g., stroke, tumor, severe traumatic brain injury); (2) missing clinical or imaging data; (3) MRI scans with severe artifacts interfering with quantitative analysis.

This study was approved by the Ethics Committee of the First Affiliated Hospital of Chongqing Medical University and was conducted in accordance with the Declaration of Helsinki. Written informed consent was obtained from all participants or their legal guardians.

### 2.2 Clinical evaluation

All subjects underwent standardized neuropsychological assessments. Global cognitive function was evaluated using the Montreal Cognitive Assessment Scale (MoCA) and the Mini-Mental State Examination (MMSE). Assessments were administered by trained neuropsychologists.

For patients, we recorded disease duration (symptom onset to MRI) and treatment duration (first immunotherapy to MRI). Acute-phase clinical severity was retrospectively assessed using the maximum modified Rankin Scale (mRS) score from initial hospitalization. CSF findings (pleocytosis >5 × 10^6^/L and anti-NMDAR antibody titer) were extracted from medical records when available (cell count data for 68/103 patients). These acute-phase laboratory measures were used for descriptive characterization of the cohort rather than as covariates in primary imaging-cognition models, given their temporal dissociation from the convalescent-phase MRI acquisition.

### 2.3 MRI data acquisition

All brain MRI scans were performed using a 3.0 Tesla clinical MRI scanner (Siemens Healthineers, Magnetom Skyra, Erlangen, Germany) equipped with a 32-channel phased-array head coil.

Three-dimensional T1-weighted magnetization-prepared rapid gradient echo (MPRAGE) images were acquired with the following parameters: repetition time (TR) = 2300 ms, echo time (TE) = 2.26 ms, inversion time (TI) = 900 ms, number of slices = 192, slice thickness = 1.0 mm, field of view (FOV) = 256 mm, voxel size = 1.0 × 1.0 × 1.0 mm³, and acquisition time = 5 min 21 s.

DTI was acquired using a single-shot echo-planar imaging sequence with the following parameters: TR *=* 5700 ms, TE = 86 ms, FOV = 240 × 240 mm², voxel size = 2.5 × 2.5 × 2.3 mm³, number of slices = 53, slice thickness = 2.3 mm, 34 non-collinear diffusion gradient directions at a single b-value of 1000 s/mm² plus 1 non-diffusion-weighted (b = 0) volume. Parallel imaging using GRAPPA with an acceleration factor of 2 and partial Fourie *r =* 6/8 were applied. The signal-to-noise ratio was approximately 40 for the b = 0 image and 15 for the diffusion-weighted images. This single-shell acquisition (b = 1000 s/mm²) was used for all DTI-derived metrics, including the standard tensor fit for ALPS directional diffusivities and FA maps.

DTI preprocessing included eddy current correction, phase-encoding distortion correction, and head motion correction. Participants with significant head motion artifacts were excluded from subsequent analyses.

### 2.4 Image data processing

#### 2.4.1 Calculation of the DTI-ALPS index

**Figure 1:**
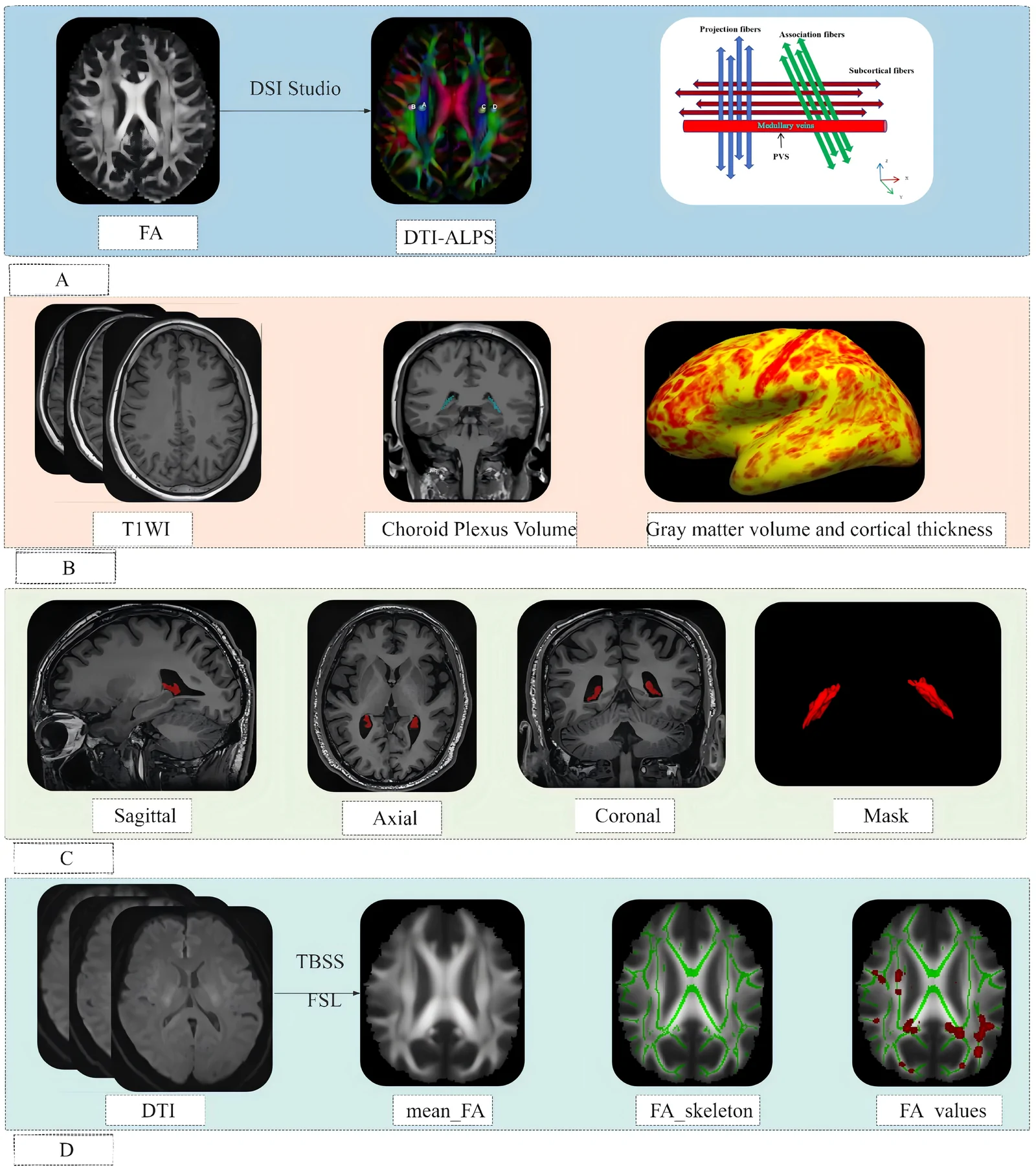
**(A)** DTI-ALPS calculation using the diffusivities of water molecules along the medullary veins in the centrum semiovale region, normalized by diffusivities along the projection and association fibers. **(B)** Automated segmentation of the choroid plexus and gray matter using FreeSurfer. Representative T1-weighted image (left), automatic segmentation of the bilateral choroid plexus (middle), and parcellation of cortical gray matter into 68 regions per hemisphere using the Desikan-Killiany atlas (right). **(C)** Manual re-delineation of the choroid plexus using ITK-SNAP when FreeSurfer segmentation was inaccurate. Sagittal, axial, and coronal views of a representative case where FreeSurfer overestimated the CP volume due to inflammation-related remodeling or adjacent artifacts. **(D)** TBSS-based white matter microstructural analysis. DTI data were preprocessed, followed by nonlinear registration to MNI space. A mean FA skeleton (green) was generated at FA threshold 0.2, and skeletonized FA maps were used for voxel-wise group comparisons. Global mean FA was extracted from all significant clusters (red) after TFCE correction.

The DTI-ALPS index, an indirect MRI marker of glymphatic function, was computed as follows (**Figure 1A**). First, DTI data were converted from Digital Imaging and Communications in Medicine (DICOM) format to the DSI Studio source (SRC) format for processing in DSI Studio (26). Data with severe motion artifacts were excluded after strict quality control. Eddy current correction and phase-encoding distortion correction were performed using built-in functions in DSI Studio. A brain mask was generated to remove non-brain tissue and improve the reconstruction and analyses. After preprocessing, DTI data were reconstructed to obtain the direction of water molecules. Whole-brain probabilistic fiber tracking was performed using the default settings in DSI Studio to confirm fiber orientation; projection fibers near the lateral ventricles mainly run along the z-axis (craniocaudal direction), while association fibers such as the superior longitudinal fasciculus run mainly along the y-axis (anterior-posterior direction).

The ALPS index was calculated from DTI data using a previously described method with minor modifications. Two board-certified neuroradiologists independently delineated four regions of interest (ROIs), each consisting of 3 × 3 contiguous voxels, on the same axial slice of the FA color map at the level of the body of the lateral ventricles. ROIs were carefully placed to avoid lesions and imaging artifacts. Two ROIs were placed in the projection fiber region, where fibers run predominantly along the z-axis; the other two ROIs were placed in the association fiber region, where fibers run mainly along the y-axis in both hemispheres. Using DTI, we separately extracted the mean diffusion coefficients along the x-, y-, and z-axes for each ROI, allowing quantitative comparisons between projection and association fibers. The calculation formulas are as follows:


$${\text{DTI-ALPS}}_{\text{index}}=\frac{{\text{D}}_{\text{x}}^{\text{proj}}/{\text{D}}_{\text{x}}^{\text{assoc}}}{{\text{D}}_{\text{y}}^{\text{proj}}/{\text{D}}_{\text{z}}^{\text{assoc}}}$$

Where ${\text{D}}_{\text{x}}^{\text{proj}}$ and ${\text{D}}_{\text{x}}^{\text{assoc}}$ represent the mean diffusion rates of projection fibers and association fibers along the x-axis, respectively; ${\text{D}}_{\text{y}}^{\text{proj}}$ denotes the mean diffusion rate of projection fibers along the y-axis; and ${\text{D}}_{\text{z}}^{\text{assoc}}$ denotes that of association fibers along the z-axis. The final DTI-ALPS index was calculated as the average of the bilateral cerebral hemispheres. Inter-observer reliability for ALPS ROI placement was assessed using the intraclass correlation coefficient (ICC). Inter-observer reliability for ALPS ROI placement was excellent (ICC > 0.95).

#### 2.4.2 Brain structure segmentation and CP volume extraction

T1-weighted images were processed using the standard FreeSurfer (v7.2.0) (27) workflow, which included motion correction, skull removal, spatial normalization, and intensity normalization. From these preprocessed images, we extracted CP volume, gray matter volume, and cortical thickness (**Figure 1B**).

CP volume: Bilateral CP was first automatically segmented using FreeSurfer’s built-in anatomical template. All automatic segmentations were independently reviewed by two experts (ICC > 0.95). When FreeSurfer segmentation was inaccurate (e.g., overestimation or underestimation due to inflammation-related CP remodeling or adjacent artifacts), manual refinement was performed using ITK-SNAP (version 3.8.0). **Figure 1C** illustrates a representative case of manual re-delineation, showing sagittal, axial, and coronal views and the final binary mask used for volume calculation. Manual corrections were performed blinded to group status. To account for individual variations in head size, the ratio of CP volume to intracranial volume (ICV)—i.e., the CP/ICV ratio—was calculated as the primary metric.

Cortical and subcortical volume: The gray matter volumes and cortical thickness of 68 bilateral brain regions were extracted using the Desikan-Killiany atlas. All segmentations were visually inspected for quality control; and no subjects were excluded due to inadequate image quality.

To control for confounding factors, covariate adjustments were performed on the volumes of the 68 brain regions, cortical thickness, and the CP/ICV ratio. A linear regression model was constructed for each measure:


$$\text{Volume}\;=b_{o}+b_{1}._{\text{age}}+\;{\text{b}}_{2}._{\text{sex}}+b_{3}._{\text{education}}+b_{4}._{\text{icv}}+\;\text{ϵ}$$

The residuals from each regression were extracted and converted into Z-scores for subsequent SCN analysis (see section 2.5).

#### 2.4.3 TBSS-based analysis of brain white matter microstructure

To compare white matter microstructure between groups, TBSS was performed using FMRIB Software Library (FSL) version 6.0 (28). The preprocessed FA maps were nonlinearly registered to the MNI-152 standard space. An average FA template was constructed, and a white matter skeleton was generated using an FA threshold of 0.2. All subjects’ FA data were then projected onto this skeleton to obtain standardized skeletonized FA maps. **Figure 1D** summarizes the TBSS analysis pipeline, including DTI preprocessing, skeleton generation, and extraction of global mean FA.

Group comparisons were performed using a randomized non-parametric permutation test (5, 000 permutations) combined with threshold-free clustering enhancement (TFCE). Multiple comparisons were controlled within the TFCE framework, and a significance threshold of *P<* 0.05 was used. All voxels showing significant FA difference between groups were merged into a single global ROI. The mean FA value within this ROI was extracted as a quantitative measure of overall white matter microstructural damage.

### 2.5 Construction of SCN and graph theory analysis

Group-level SCNs were constructed separately for gray matter volume and cortical thickness, based on the 68 brain regions (Desikan-Killiany atlas) after covariate adjustment (as described in section 2.4.2).

Network construction: For each group (anti-NMDAR encephalitis and HCs), Pearson correlation coefficients were calculated between the adjusted volumes of all pairs of the 68 regions, resulting in a 68 × 68 correlation matrix. Only positive correlations were retained; negative correlations were discarded, and the diagonal elements were set to zero.

Thresholding: A proportional thresholding method (sparsity/density) was applied with a threshold range of 0.05 to 0.20 (step size 0.01), generating 16 binarized networks for each group. This sparsity range covers the typical interval for small-world brain networks;HCs exhibited σ > 1, consistent with canonical human brain network properties, while patients showed reduced σ, indicating impaired small-worldness. This approach ensures comparable small-world properties across different network densities.

Graph theory metrics: Global and nodal graph metrics were computed using the MATLAB Brain Connectivity Toolbox (BCT; https://sites.google.com/site/bctnet/). The following global metrics were analyzed: clustering coefficient (Cp), characteristic path length (Lp), global efficiency (E _global), local efficiency (E _local), modularity (Q), assortativity, and the small-world property (σ). The nodal metric was betweenness centrality, which reflects the importance of a node in information flow across the network. Because SCNs were constructed at the group level, these network analyses were exploratory and descriptive. Individual-level network metrics were not derived, precluding their use in subsequent mediation analyses.

### 2.6 Statistical analysis

Statistical analyses were conducted using Python (SciPy, Statsmodels), R (v4.3.3), and MATLAB (R2021b). All tests were two-tailed, and a false discovery rate (FDR)-corrected *q* value< 0.05 using the Benjamini-Hochberg (BH) procedure was considered statistically significant, unless otherwise specified.

#### 2.6.1 Descriptive statistics and intergroup comparisons

Descriptive statistics were employed to summarize the demographic, clinical, and imaging characteristics of the study population. Group comparisons and subsequent correlation analyses were adjusted for age, sex, and years of education in both groups; for patients, additional adjustments were made for disease duration and treatment duration to minimize clinical confounding. Anti-NMDAR antibody titers, although recorded at diagnosis, were not included as covariates in the primary models due to their temporal mismatch with convalescent-phase imaging metrics (mean 7.6 months post-onset), and to avoid overparameterization given the limited sample size. The normality of continuous variables was assessed using the Shapiro-Wilk test. Normally distributed data were presented as the mean ± standard deviation (SD), whereas non-normally distributed data were reported as the median and interquartile range (IQR). Categorical variables were expressed as counts (percentages).

Group differences in continuous variables were assessed using two-sample t-tests for normally distributed data or Mann-Whitney U tests for non-normally distributed data. Categorical variables were compared using the chi-squared test or Fisher’s exact test, as appropriate. Specifically, to account for the non-normal distribution of cognitive scores and certain imaging metrics, non-parametric tests were primarily utilized for intergroup comparisons.

Global network metrics (area under the curve, AUC) were compared between groups using permutation tests (1, 000 repetitions). Node-level betweenness centrality was similarly compared using permutation tests. To control for the FDR arising from multiple comparisons, all P-values were adjusted using the BH method, and a q-value< 0.05 was considered statistically significant. To assess the robustness of our findings against potential nonlinear age-related confounding, we performed sensitivity analyses for the CP/ICV group comparison using three covariate adjustment strategies: (1) linear adjustment for age; (2) quadratic adjustment (age + age²); and (3) cubic adjustment (age + age² + age³). The consistency of the group effect across these models was examined to confirm the stability of the CP/ICV difference between patients and controls.

#### 2.6.2 Correlation analysis

All correlation analyses were performed exclusively within the patient group (n = 103) to avoid Simpson-type confounding from pooling patients and controls (who differ systematically in both cognition and imaging metrics).Pearson or Spearman partial correlation analyses were conducted, controlling for age, sex, and years of education as covariates. Of note, the unadjusted group comparisons in **Table 1** serve descriptive purposes; all inferential analyses (correlations, mediation) incorporate the above covariates to minimize confounding. For the patient group, disease duration and treatment duration were additionally included as covariates to account for clinical confounding factors. The choice between Pearson and Spearman methods was determined by the normality of the specific variables involved.

**Table 1:** Demographics, clinical characteristics, white matter microstructure, and cognitive characteristics of the anti-NMDAR encephalitis group and the HCs group.

Variable	HC group (n=97)	Anti-NMDAR encephalitis group (n=103)	*P*-value	FDR-corrected q-value
Age (years)	40.835 ± 8.616	37.320 ± 14.897	0.0235^b^	_
Sex (Male/Female)	40/57	48/55	0.5344^c^	_
Education (years)	12.567 ± 3.262	10.437 ± 2.939	<0.0001^b^	_
MoCA score	26.959 ± 2.553	22.126 ± 2.519	<0.0001^b^	_
MMSE score	29.412 ± 0.921	24.039 ± 2.086	<0.0001^b^	_
CP/ICV ratio	0.071 ± 0.019	0.080 ± 0.026	0.0090^b^	0.0135
Total CP volume (mm³)	1043.261 ± 299.552	1116.799 ± 370.164	0.1166^b^	0.1399
Total intracranial volume (ICV, mm³)	1465119.127 ± 144961.121	1398954.926 ± 244373.506	0.0945^b^	_
FA value	0.597 ± 0.099	0.506 ± 0.052	<0.0001^b^	<0.0001
Left ALPS index	1.489 ± 0.178	1.361 ± 0.196	<0.0001^b^	<0.0001
Right ALPS index	1.483 ± 0.174	1.335 ± 0.183	<0.0001^b^	<0.0001
Mean DTI-ALPS index (left + right)/2	1.486 ± 0.140	1.348 ± 0.143	<0.0001^b^	<0.0001
Acute-phase clinical indicators				
Time to diagnosis (days)	_	16.3 ±13.4	_	_
Time to first immunotherapy (days)	_	15.4 ±13.5	_	_
Length of hospitalization (days)	_	28.9 ±17.8	_	_
Interval from onset to MRI (months)	_	7.5 ± 2.3	_	_
mRS maximum score(median [IQR], range)	_	4 (3–5, 2–5)	_	_
CSF pleocytosis (>5×10^6^/L), n (%)	_	50/68(73%)^d^	_	_
CSF antibody titer	_	1:32 (1:10–1:32,range 1:10–1:100)^e^	_	_
Acute-phase immunotherapy (n, %)				
Corticosteroids	_	86/103 (83.5%)	_	_
IVIG	_	35/103 (34.0%)	_	_
Second-line immunotherapy	_	8/103 (7.8%)	_	_

Data are presented as mean ± standard deviation (SD) unless otherwise indicated.

a P values were derived from two-sided tests: two-sample t-test for normally distributed continuous variables, Mann–Whitney U test for non-normally distributed continuous variables, and chi-square test for categorical variables.

b P value from Mann-Whitney U test.

c P value from chi-squared test.

d CSF pleocytosis data were available for 68 of 103 patients due to the retrospective study design.

e Serum or CSF anti-NMDAR antibody titer at diagnosis; reported as median (IQR, range).

The mRS maximum score reflects peak acute-phase severity, not the score at MRI acquisition (post-acute phase; mean 7.5 ± 2.3 months after onset). Unadjusted P-values are reported for baseline demographics and cognitive scores. FDR-corrected q-values (Benjamini–Hochberg) are presented for imaging metrics (DTI-ALPS: 3 indices; CP/ICV and FA: single measures). ICV was not FDR-corrected as it served as a normalization denominator rather than a primary outcome. All subsequent correlation and mediation analyses were adjusted for age, sex, and education (see Methods Sections 2.6.2 and 2.6.3). ‘—’ indicates not applicable or not tested.

Due to the shared methodological basis of DTI-ALPS and FA (both derived from diffusion tensor imaging), the correlation between these two specific white matter integrity indices was examined without multiple comparison correction to avoid over-adjustment. All other correlation analyses involving cognitive scores (MoCA, MMSE) and brain network metrics were corrected for multiple comparisons using the BH method (*q<* 0.05).

#### 2.6.3 Exploratory mediation analysis

Mediation analysis was performed as an exploratory, hypothesis-generating approach using the Bootstrap resampling method (5, 000 iterations) to examine whether global mean FA (the white matter microstructural damage index) mediated the association between each CSF-related marker (DTI-ALPS index or CP/ICV ratio) and cognitive scores (MoCA, MMSE). Cross-sectional data cannot establish the temporal sequence required for mediation; therefore, our findings reflect statistical associations, not causal pathways.Brain network topology was not included in this mediation analysis because SCNs were constructed at the group level, precluding individual-level network metrics. All variables were standardized to Z-scores, and age, sex, and education were included as covariates. The indirect effect was estimated by bootstrap, and its 95% confidence interval (CI) was calculated; a CI that did not include zero was considered evidence of significant mediation. The proportion mediated (indirect effect/total effect) was calculated for significant mediation effects.

#### 2.6.4 Sensitivity/power analysis for mediation

To determine the minimum indirect effect detectable at the achieved sample size, a Monte Carlo simulation-based sensitivity analysis was conducted. For each candidate standardized indirect effect size (ab ranging from 0.03 to 0.40), 2, 000 simulated datasets were generated under a mediation model with 3 covariates, and empirical power was computed as the proportion of simulations in which the bootstrap 95% CI excluded zero. The minimum detectable indirect effect was defined as the smallest ab at which power ≥ 0.80.

## 3 Results

### 3.1 Demographic and clinical characteristics of participants

Baseline demographics, cognitive, CSF-related markers, and white matter microstructure characteristics are presented in **Table 1**.

A total of 103 patients with anti-NMDAR encephalitis (48 males, 55 females; mean age 37.32 ± 14.90 years) and 97 HCs (40 males, 57 females; mean age 40.84 ± 8.62 years) were enrolled. There was no significant difference in sex distribution between the two groups (*P* = 0.534). However, the patient group had significantly fewer years of education than the control group (*P<* 0.0001), and age differed between groups (*P* = 0.0235). All subsequent analyses were therefore adjusted for age, sex, and years of education as covariates.

In the patient group, the mean time from symptom onset to diagnosis was 16.3 ± 13.4 days, and the mean time to first immunotherapy was 15.4 ± 13.5 days. The average length of hospitalization was 28.9 ± 17.8 days, and the interval from onset to MRI was 7.5 ± 2.3 months, indicating that all patients were in the post-acute/recovery phase at the time of imaging. The acute-phase mRS maximum score was median 4 (IQR 3–5, range 2–5). CSF pleocytosis (>5 × 10^6^/L) was present in 50/68 (73%) patients with available CSF data, and the CSF antibody titer was median 1:32 (IQR 1:10–1:32, range 1:10–1:100). Regarding acute-phase immunotherapy, 86 patients (83.5%) received corticosteroids, 35 (34.0%) received intravenous immunoglobulin (IVIG), and 8 (7.8%) received second-line immunotherapy. The between-group comparisons shown in **Table 1** present both unadjusted P-values and FDR-corrected q-values for imaging metrics where multiple comparisons were performed. For demographics and cognitive scores, only unadjusted P-values are reported as these were single comparisons. All subsequent correlation and mediation analyses were performed using covariate-adjusted models.

In neuropsychological assessments, the patient group showed significantly lower cognitive performance compared to controls, as reflected by lower MoCA scores (22.13 ± 2.52 vs. 26.96 ± 2.55, *P<* 0.0001) and lower MMSE scores (24.04 ± 2.09 vs. 29.41 ± 0.92, *P<* 0.0001).

Regarding imaging metrics, the patient group exhibited a significantly higher CP/ICV ratio (0.080 ± 0.026 vs. 0.071 ± 0.019, *q* = 0.0135), indicating choroid plexus remodeling. The bilateral DTI-ALPS indices were significantly lower in patients than in controls (left: 1.361 ± 0.196 vs. 1.489 ± 0.178, *q<* 0.0001; right: 1.335 ± 0.183 vs. 1.483 ± 0.174, *q<* 0.0001), consistent with impaired glymphatic function. The mean (left + right) DTI-ALPS index was also significantly lower in patients (1.348 ± 0.143 vs. 1.486 ± 0.140, *q* < 0.0001). In addition, the global mean FA (derived from TBSS) was markedly reduced in patients compared to controls (0.506 ± 0.052 vs. 0.597 ± 0.099, *q* < 0.0001), indicating widespread white matter microstructural damage.

**Figure 2:**
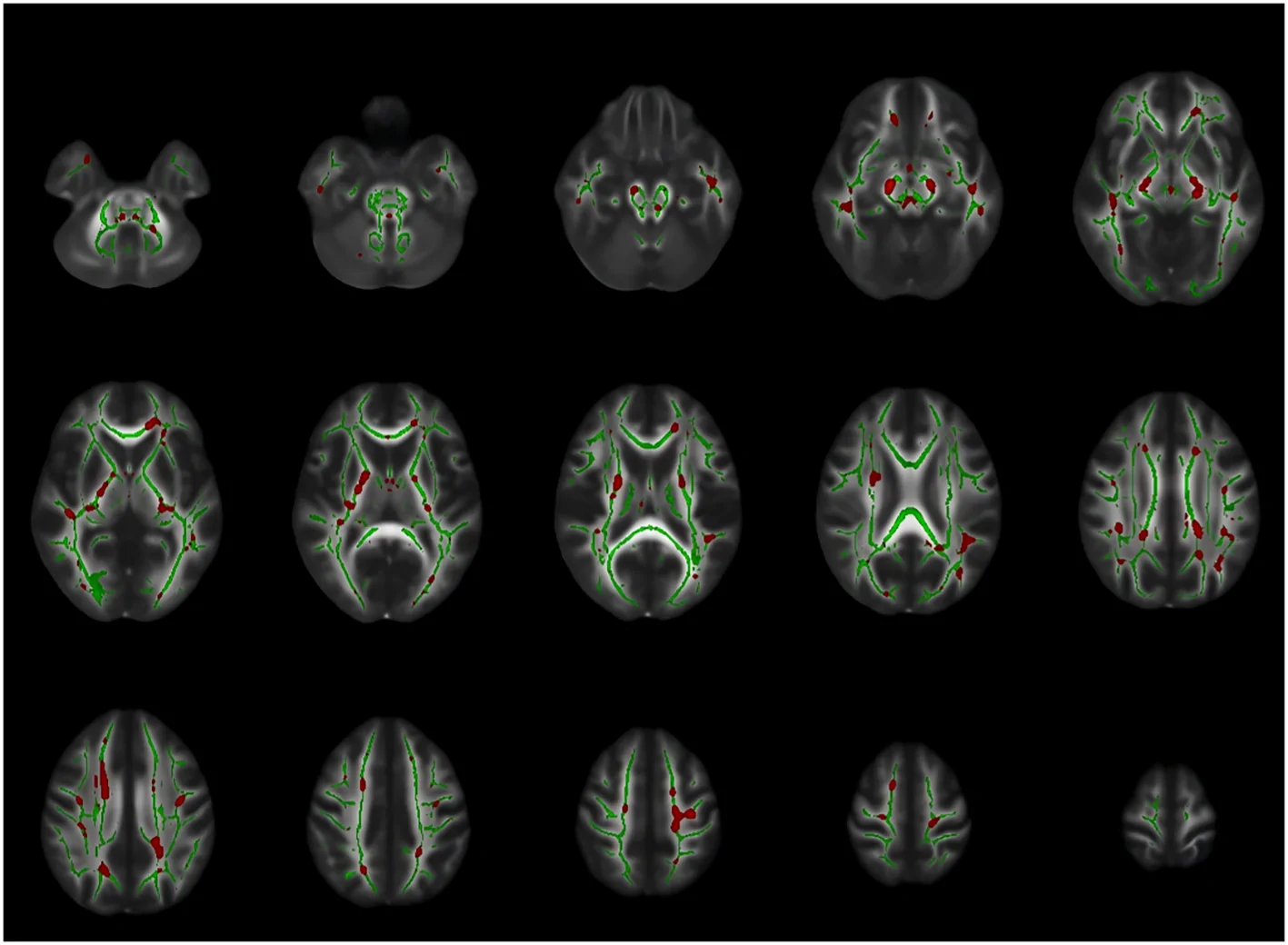
TBSS analysis of white matter FA differences between anti-NMDAR encephalitis patients and HCs. Background: standard FA template; green: whole-brain white matter skeleton; red: regions with significantly reduced FA in the patient (all clusters combined, *P<* 0.05, TFCE-corrected). The global mean FA was extracted from this significant mask for correlation analysis with the DTI-ALPS index.

Global mean FA, derived from TBSS, was markedly reduced in patients compared to controls (0.506 ± 0.052 vs. 0.597 ± 0.099, FDR-corrected *q<* 0.0001), indicating extensive white matter microstructural damage. Voxel-wise TBSS analysis further revealed widespread FA reduction in patients across multiple fiber tracts (*q<* 0.05; TFCE-corrected). Affected regions included core tracts involved in cognition, emotion, and neural transmission: bilateral anterior thalamic radiations, posterior limb of the internal capsule, corticospinal tract, body and callosal portion of the corpus callosum, superior longitudinal fasciculus, inferior fronto-occipital fasciculus, and cingulate tract (**Figure 2**). No significant FA increases were observed.

To evaluate the potential impact of nonlinear age-related confounding on the CP/ICV finding, sensitivity analyses were performed using quadratic and cubic age adjustments. The group effect remained positive and significant across all models (linear: coefficient = 0.0096, *P* = 0.0036; quadratic: coefficient = 0.0077, *P* = 0.0329; cubic: coefficient = 0.0083, *P* = 0.0214), confirming the robustness of the CP/ICV elevation in patients.

### 3.2 Correlation analysis

**Figure 3:**
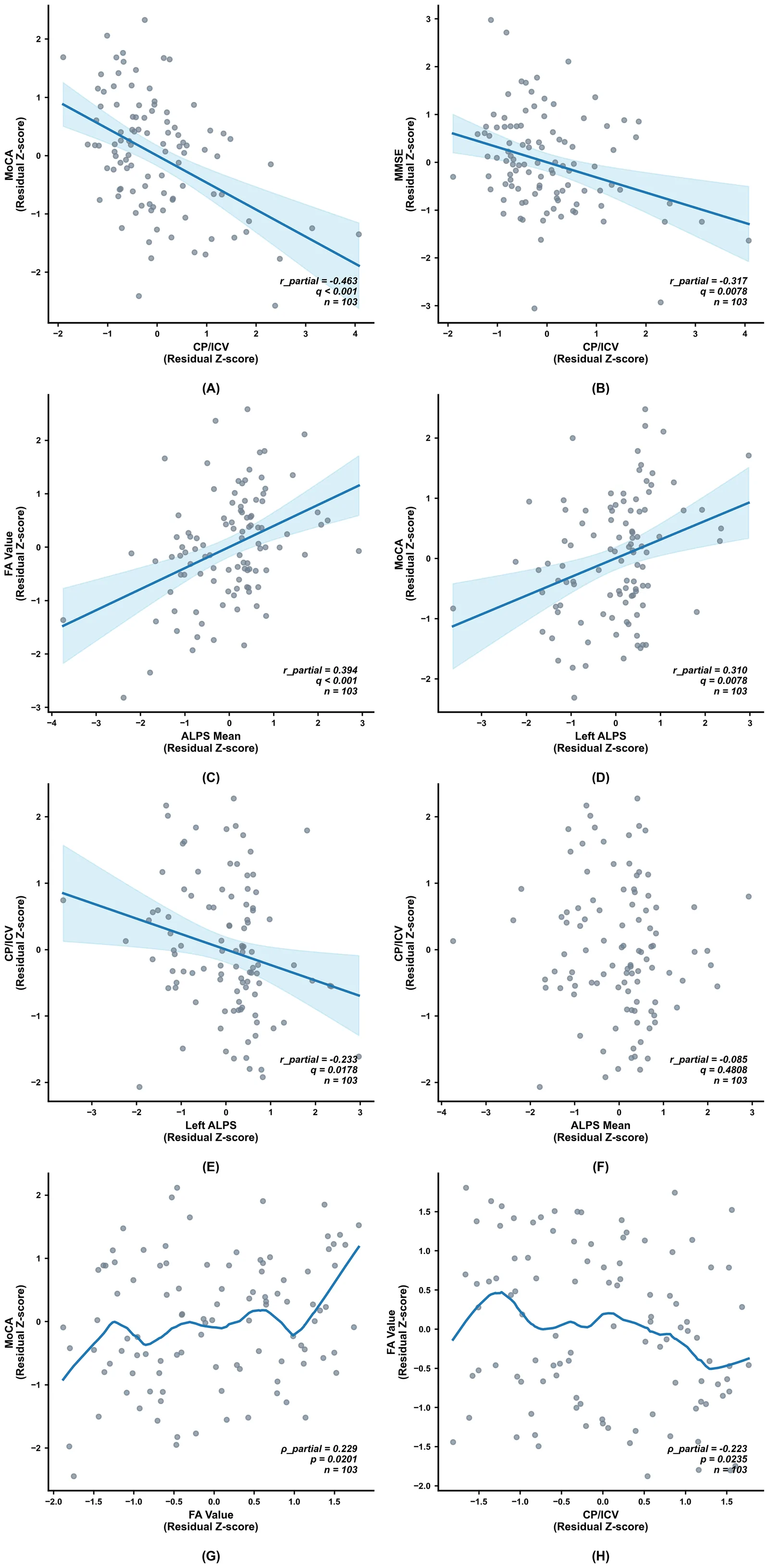
Partial correlations among the DTI-ALPS index, CP/ICV ratio, global FA, and cognitive scores in anti-NMDAR encephalitis patients. Black solid lines: linear regression fit for Pearson correlations; light blue shaded areas: 95% confidence interval; blue curves: LOESS smoothing for Spearman correlations. **(A)** CP/ICV ratio vs. MoCA score; **(B)** CP/ICV ratio vs. MMSE score; **(C)** Mean ALPS index vs. global FA; **(D)** Left ALPS index vs. MoCA score; **(E)** Left ALPS index vs. CP/ICV ratio; **(F)** Mean ALPS index vs. CP/ICV ratio; **(G)** global FA vs. MoCA score; **(H)** CP/ICV ratio vs. global FA. Panels **(A–F)** report FDR-corrected q values; **(G, H)** report uncorrected p values, as these represent exploratory between-marker associations.

All correlation analyses reported in this section were performed exclusively within the patient group (n = 103) using covariate-adjusted partial correlation models. After controlling for age, sex, and years of education, Pearson and Spearman correlation analyses were performed to examine associations among CSF-related markers, white matter microstructure, and cognitive scores. For the CP/ICV-cognition analyses, the DTI-ALPS index, global FA, ICV, and total CP volume were additionally controlled to isolate the unique contribution of choroid plexus structural remodeling to cognitive outcomes, independent of glymphatic function, white matter microstructural integrity, and brain volume. Spearman rank correlation was applied to non-normally distributed variables, while Pearson correlation was used for normally distributed pairs. All reported *q*-values were corrected for multiple comparisons using the BH method. All correlation results are shown in **Figure 3**.

CSF-related markers and cognition. The CP/ICV ratio showed the strongest association with cognitive impairment, demonstrating robust negative correlations with both the MoCA (**Figure 3A**; partial *r* = -0.463, *q* < 0.001) and MMSE scores (**Figure 3B**; partial *r* = -0.317, *q* = 0.0078). Among the DTI-ALPS indices, the left-sided ALPS index showed a significant positive correlation with MoCA (**Figure 3D**; partial *r =* 0.310, *q =* 0.0078), whereas the mean DTI-ALPS index did not reach significance after FDR correction (partial rho = 0.149, *q =* 0.2432), and neither did the right-sided ALPS index (partial rho = 0.063, *q =* 0.5794). No ALPS indices were significantly correlated with MMSE scores after FDR correction.

Associations between glymphatic surrogate and CP remodeling. The left-sided DTI-ALPS index showed a significant negative partial correlation with the CP/ICV ratio (**Figure 3E**; partial *r* = −0.233, *p* = 0.0178), whereas neither the mean DTI-ALPS index (**Figure 3F**; partial *r* = −0.085, *p* = 0.4808) nor the right-sided DTI-ALPS index (partial *ρ* = −0.160, *p* = 0.1064) reached significance. This hemispheric asymmetry suggests that glymphatic function in the left hemisphere may be preferentially associated with choroid plexus morphological remodeling.

White matter microstructure and its association. Global FA showed a strong positive correlation with the mean DTI-ALPS index (**Figure 3C**; partial *r* = 0.394, *q* < 0.001). Regarding cognitive function, global FA demonstrated a modest but non-significant positive correlation with the MoCA score after FDR correction (**Figure 3G**; partial *ρ* = 0.229, *p* = 0.020, *q* = 0.0648), and was not significantly correlated with MMSE performance (partial *ρ* = 0.141, *q* = 0.2443). A weaker negative correlation was observed between global FA and the CP/ICV ratio (**Figure 3H**; partial *ρ* = –0.223, *p* = 0.024, *q* = 0.0648).

Additionally, exploratory partial correlation analysis was performed to assess the association between CP/ICV and clinical severity evaluated by the mRS. After controlling for age, sex, and education, no significant correlation was found between CP/ICV and mRS scores (partial *r* = –0.058, *p* = 0.560, n = 103; zero-order *r* = -0.068, *p* = 0.496, ns).

### 3.3 Exploratory analysis of brain structural network topology

#### 3.3.1 Cortical thickness network analysis

For the cortical thickness-based SCN, no significant group differences survived multiple comparison correction. Although unadjusted comparisons showed nominally significant differences in clustering coefficient (Cp, *P* = 0.048), global efficiency (*E_global*, *P* = 0.042), and assortativity (*P* = 0.035), none remained significant after Benjamini-Hochberg FDR correction (all *q* > 0.315). At the node level, the patient group showed betweenness centrality in regions such as the left insula, fusiform gyrus, middle frontal gyrus, and right parahippocampal gyrus in unadjusted analyses, but these findings also did not survive FDR correction. Overall, the cortical thickness network did not provide evidence of robust topological alterations in anti-NMDAR encephalitis.

#### 3.3.2 Cortical volume network analysis

In contrast, the cortical volume-based SCN revealed robust topological remodeling in the patient group. Permutation test showed significant differences in *Cp*, *Q*, and *E_global* (all permutation *P<* 0.001), which remained significant after FDR correction (*q<* 0.001). Specifically, patients exhibited increased *Cp* and *Q* and decreased *E_global*, indicating enhanced local segregation and reduced global integration. Characteristic *Lp* and assortativity showed nominal differences (*P* = 0.019 and *P* = 0.041, respectively), but these did not survive FDR correction (*q* = 0.057 and *q* = 0.082). At the node level (at a density of 0.12, unadjusted), patients showed significantly increased betweenness centrality in the left middle frontal gyrus, left superior parietal lobule, right precuneus, right middle temporal gyrus, right precentral gyrus, and right supramarginal gyrus.

#### 3.3.3 Comparison and conclusion

**Figure 4:**
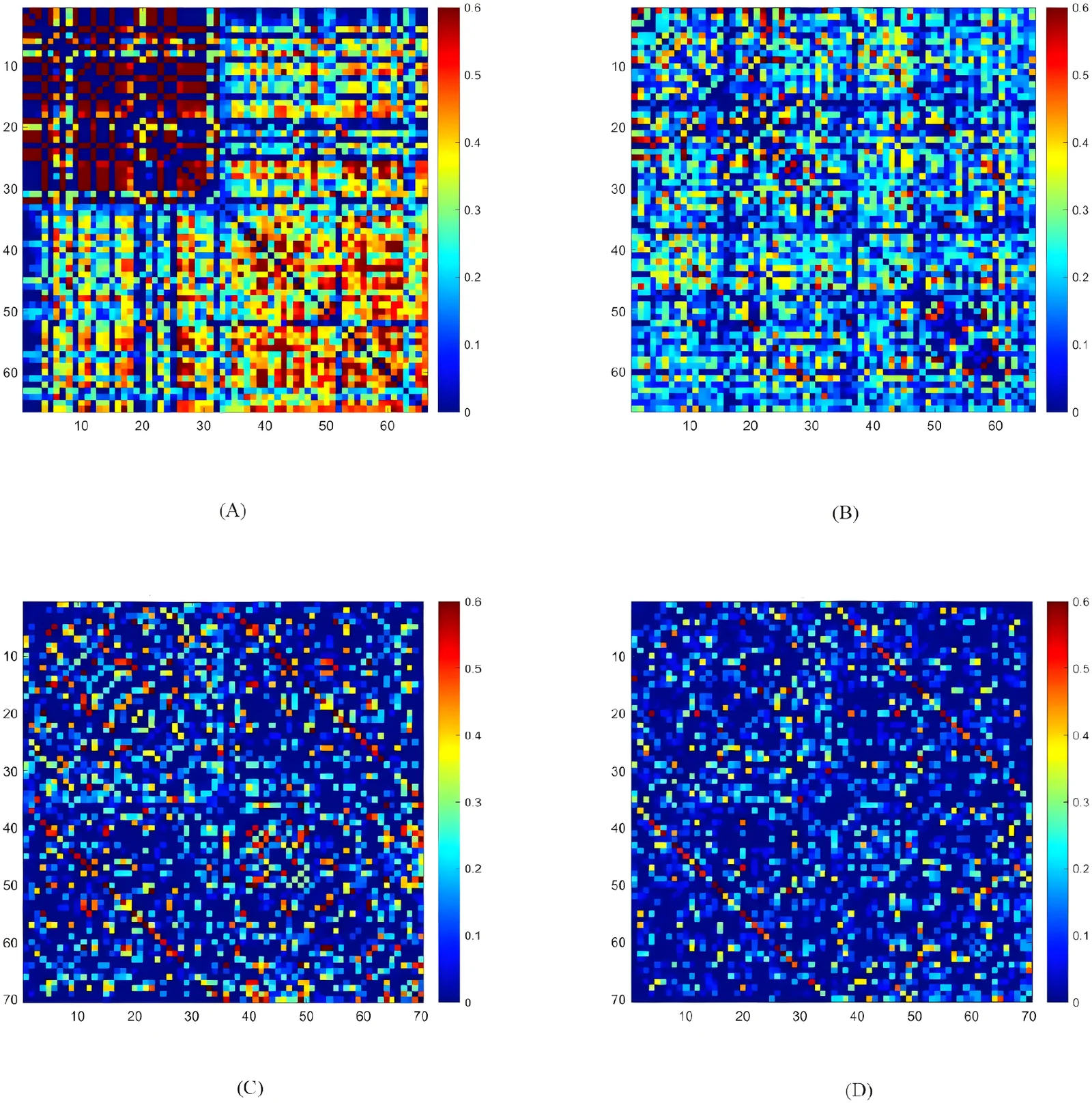
Structural covariance matrices of cortical volume and thickness in anti-NMDAR encephalitis patients and healthy controls. **(A)** Cortex volume matrix, patients; **(B)** Cortex volume matrix, HC; **(C)** Cortex thickness matrix, patients; **(D)** Cortical thickness matrix, HC. Matrices were computed using Pearson correlations across 68 cortical regions; only positive correlations are shown. The color bar indicates correlation strength: red = strong positive correlation, blue = weak/no correlation.

The structural covariance matrices for both volume and thickness are displayed in **Figure 4**. In summary, robust topological reorganization (increased *Cp* and *Q*, decreased *E_global*) was observed only in the cortical volume network, whereas the cortical thickness network showed no significant group differences after correction. These findings indicate that cortical volume-based SCN shows robust topological alterations in anti-NMDAR encephalitis, while cortical thickness-based SCN does not. This discrepancy may reflect that cortical volume is more sensitive to inflammation-related brain atrophy, whereas cortical thickness changes are subtler in this cohort.

### 3.4 Association between CSF-related marker and cognitive function

Based on the correlation findings between the CP/ICV ratio, the DTI-ALPS index, and cognitive function, this study conducted mediation analysis using 5, 000 bootstrap resampling iterations. All mediation models included age, sex, and years of education as covariates. After controlling for age, gender, and years of education, the study examined the potential mediating pathways of the CP/ICV ratio and the DTI-ALPS index on cognitive function, employing brain network topology metrics and white matter microstructural damage indicators (global mean FA) as mediation variables. The potential mediating role of brain network topology metrics was not examined in the current cross-sectional analysis due to the group-level nature of SCN construction, and remains to be tested in future individual-level network studies.

Sensitivity analysis showed adequate power for indirect effects ≥ 0.13, but the observed effect (0.075) fell below this, warranting caution. Bootstrap mediation (5, 000 resamples; covariates: age, sex, education) revealed that global FA significantly mediated the DTI-ALPS-MoCA association (indirect effect = 0.075, 95% CI [0.011, 0.152]; proportion mediated = 70.57%), but not ALPS–MMSE, CP/ICV–MoCA, or CP/ICV–MMSE. Given the strong methodological collinearity between DTI-ALPS and FA (partial *r* = 0.394) and the modest power for this small effect, this mediation likely reflects shared diffusion variance rather than a distinct biological pathway. The cross-sectional design precludes causality. Nonetheless, the result suggests white matter integrity partially accounts for the ALPS–cognition link. The CP/ICV–cognition association, however, appears independent of global FA, implying other pathways.

## 4 Discussion

This study used multimodal MRI to systematically investigate glymphatic function (via DTI-ALPS as a surrogate marker) and CP structural remodeling (via CP/ICV ratio), white matter microstructure, topological characteristics of brain morphological covariance networks, and their associations with cognitive function in the anti-NMDAR encephalitis group. The results showed that the patients exhibited distinct CSF-related marker abnormalities, manifested as significantly decreased DTI-ALPS index and elevated relative CP volume (CP/ICV ratio). TBSS-based whole-brain analysis revealed extensive white matter microstructural damage with significantly reduced global FA. Meanwhile, brain morphological covariance networks in patients showed typical topological reorganization characterized by enhanced local connectivity and reduced global integration. Further exploratory mediation analysis revealed that global FA significantly mediated the DTI-ALPS–MoCA association, but did not mediate the CP/ICV–cognition associations, suggesting differential mechanistic pathways.

This study provides evidence of significant CSF-related impairment in anti-NMDAR encephalitis during the post-acute phase, extending previous observations beyond the initial acute stage. Compared with the HCs, patients showed bilaterally decreased DTI-ALPS indices, suggesting severe impairment of perivascular CSF circulation, compromising interstitial waste clearance. The brain glymphatic system is responsible for clearing metabolic wastes, and its function depends on intact CSF circulation and AQP4-mediated fluid exchange (8, 29). This abnormality is likely driven by multiple pathological mechanisms: inflammatory obstruction of perivascular spaces, disruption of blood-brain barrier integrity, structural damage to astrocytic end feet, and loss of AQP4 polarity (30). Anti-NMDAR encephalitis-related autoantibodies further disrupt neuron-glia interactions and exacerbate CSF-related abnormalities (31). Direct evidence of humoral immunity activation comes from recent CSF proteomics showing elevated B cell-associated proteins IGLC2, MZB1, and CD79B in pediatric anti-NMDAR encephalitis (32).

Correlation analysis revealed that the left-sided DTI-ALPS index exhibited only a weak but significant positive correlation with MoCA scores, suggesting a modest association between glymphatic function and global cognition. In contrast, the CP/ICV ratio—which was significantly elevated in patients—emerged as the predominant CSF correlate of cognitive dysfunction. This CP enlargement likely reflects inflammation-mediated choroid plexus hypertrophy, barrier disruption, and heightened immune cell trafficking into the CNS (3, 30), consistent with its role as a key gateway for peripheral immune entry and a structural indicator of intrathecal immune activation (33).

Notably, this CP/ICV elevation was observed despite the patient group being significantly younger than controls, and sensitivity analyses using nonlinear age adjustments confirmed its robustness. Given that CP volume normally increases with age, the observed higher CP/ICV in younger patients underscores the dominant effect of neuroinflammation on choroid plexus remodeling. Critically, the CP/ICV ratio showed strong negative correlations with both MoCA and MMSE, reinforcing its potential utility as a non-invasive marker for assessing inflammatory burden and predicting cognitive prognosis in anti-NMDAR encephalitis.

Extensive white matter microstructural damage was also observed in our patients, replicating prior reports (34). TBSS-based whole-brain white matter analysis revealed significantly decreased FA in major fiber tracts, including bilateral anterior thalamic radiation, internal capsule, corticospinal tract, corpus callosum, and cingulum bundle, indicating impaired white matter fiber integrity and myelin structure. However, the key novel finding lies not in white matter damage per se, but in its differential relationship with the two CSF-related markers. Bootstrap mediation revealed that global FA significantly mediated the ALPS–MoCA association, though this effect should be interpreted cautiously given the shared diffusion basis of both metrics—their strong correlation is inherently expected and likely reflects common methodological dependence on tissue microstructure rather than a causal link. Consequently, white matter damage and CSF-related abnormalities may represent parallel rather than causally sequential pathological processes. In contrast, the CP/ICV ratio—the strongest correlate of cognitive impairment—was not mediated by FA, suggesting that choroid plexus remodeling affects cognition through non-white-matter routes.

This pattern diverges from Alzheimer’s disease (AD), where glymphatic dysfunction mediates amyloid-related white matter damage via a genuine causal pathway (11, 14). In anti-NMDAR encephalitis, the apparent ALPS–FA–cognition link is largely methodological in nature, while the dominant CSF correlate—choroid plexus remodeling—operates through neuroinflammation and immune cell infiltration (19). Furthermore, AQP4 expression is chronically downregulated in AD, whereas anti-NMDAR encephalitis induces acute loss of AQP4 polarity with greater recovery potential. These distinctions caution against directly translating neurodegenerative disease models to acute autoimmune brain injury.

At the brain network level, our exploratory analysis of group-level morphological covariance networks revealed a topological pattern of enhanced local connectivity and reduced global integration in patients. Specifically, cortical volume-based SCN showed increased clustering coefficient and modularity, and decreased global efficiency. This pattern, also observed in complete thoracic spinal cord injury and schizophrenia, has been hypothesized to reflect compensatory reorganization after acute brain injury (35). However, because SCNs were constructed at the group level, these findings are descriptive and cannot be directly linked to individual-level cognitive performance or used in mediation analyses. Future studies using individual-level morphological network approaches (e.g., single-subject morphological similarity networks) or functional connectivity data are needed to test whether network reorganization mediates the relationship between CSF-related markers and cognitive impairment.

The observed increase in modularity and clustering may reflect inflammation-induced synaptic reorganization or reactive gliosis (36). In particular, increased modularity could represent functional segregation that limits the spread of pathological activity, a phenomenon observed in focal epilepsy. Whether these topological changes are reversible following immunotherapy remains unknown. Node betweenness centrality analysis revealed increased centrality in the left middle frontal gyrus, left superior parietal lobule, right precuneus, right middle temporal gyrus, right precentral gyrus, and right supramarginal gyrus in patients, suggesting compensatory enhancement of frontal and parietal hubs. Nodes related to the default mode network and limbic system may be particularly vulnerable, and network abnormalities in these regions could directly contribute to cognitive impairment (16). Of note, because the SCNs were constructed at the group level, we did not perform mediation analyses with network topological metrics; thus, the potential mediating role of network reorganization between CSF-related markers and cognition remains to be tested in future individual-level network studies.

A notable finding of this study was that the two CSF-related markers showed distinct mediation patterns in their associations with cognition. The ALPS–cognition link was partially mediated by global FA, though this likely reflects shared diffusion variance rather than a genuine pathway; in contrast, the CP/ICV–cognition relationship was not mediated by white matter integrity. This pattern can be explained from two aspects. First, methodological constraints limit the interpretability of the ALPS–FA–cognition mediation. DTI-ALPS and FA are both derived from diffusion signals in white matter, and their inherent collinearity makes it difficult to distinguish genuine mediation from shared variance—a concern reinforced by the small indirect effect size observed. Second, and more importantly, the CP/ICV ratio—the dominant correlate of cognitive impairment—exerts its influence through pathways independent of white matter integrity. Its association with cognition persisted unattenuated after accounting for FA, suggesting that choroid plexus remodeling contributes to cognitive dysfunction primarily through neuroinflammation and immune cell trafficking rather than via microstructural white matter damage.

From the perspective of clinical translation, our findings have important practical value: the DTI-ALPS index and CP/ICV ratio can serve as non-invasive multimodal imaging biomarkers for evaluating disease severity, monitoring treatment response, and assessing cognitive prognosis in anti-NMDAR encephalitis. Accumulating evidence indicates that even when conventional functional scales suggest good recovery, many patients suffer from persistent cognitive-psychiatric symptoms—including memory deficits, fatigue, anxiety, and depression—that profoundly impair quality of life and social reintegration (37). Clinically, combined assessment of ALPS and CP/ICV may facilitate early identification of patients at high risk for cognitive impairment and monitoring of immunotherapy efficacy; therapeutically, while speculative, anti-inflammatory interventions targeting the choroid plexus may be considered as future research directions to improve CSF-related function and alleviate cognitive deficits, provided they are validated in preclinical models and mechanistic studies. In addition, CSF biomarkers such as soluble CD146 (sCD146), C-X-C motif chemokine ligand 13 (CXCL13), and cellular inhibitor of apoptosis protein-1 (cIAP-1) have been shown to reflect BBB disruption and neuroinflammation, with their levels correlating with disease severity and long-term prognosis, thereby providing complementary information to strengthen disease monitoring (38, 39). The distinct mediation patterns observed for the two CSF-related markers suggest that therapies aimed at alleviating neuroinflammation—such as anti-inflammatory modulation of the choroid plexus—might directly improve cognitive outcomes, particularly through pathways independent of white matter integrity, whereas white matter damage and network reorganization may require additional or parallel interventions such as cognitive rehabilitation.

This study has several limitations. First, the DTI-ALPS index is an indirect glymphatic surrogate confounded by white matter microstructural properties, as reflected by its correlation with FA. Although our near-isotropic acquisition minimized partial volume effects, ALPS was not validated against CSF biomarkers and relies on single-slice ROI placement; future multi-shell or high-angular-resolution diffusion imaging may improve specificity. Second, the cross-sectional and retrospective design (spanning 8 years) precludes causal inference. Although we have now reported acute-phase mRS maximum scores, CSF pleocytosis rates, and antibody titers (**Table 1**), these reflect peak acute-phase status rather than the post-acute time point of MRI acquisition (mean 7.5 months post-onset); moreover, mRS and CSF pleocytosis were not available for all patients due to variable documentation practices across the enrollment period, limiting assessment of stage-dependent reversibility or treatment response. Third, SCNs were constructed at the group level, preventing individual-level mediation analyses; individual-based morphological networks are needed to test whether topology mediates CSF-cognition relationships. Future longitudinal studies with serial MRI scans starting from the acute phase are needed to delineate the temporal evolution of CSF dysfunction, white matter damage, and their relationships with cognition. Future large-sample, longitudinal, antibody-stratified multimodal imaging studies are needed to validate our findings, clarify the temporal evolution of pathology, and explore subtype-specific therapeutic targets.

## 5 Conclusion

This multimodal MRI study demonstrated that patients with post-acute anti-NMDAR encephalitis exhibit concurrent impairment in glymphatic function, CP structural remodeling, widespread white matter microstructural damage, and topological reorganization of brain networks. Among CSF-related markers, the CP/ICV ratio is the strongest correlate of cognitive dysfunction, and its association operates independently of white matter integrity. In contrast, the ALPS-cognition link, while partially mediated by global FA, is best interpreted as reflecting shared diffusion variance rather than a distinct causal pathway—a distinction that underscores the primacy of CP/ICV as a clinically meaningful biomarker. The potential mediating role of network topological alterations remains untested due to the group-level construction of SCNs construction and awaits validation in future individual-level morphological or functional connectivity studies. Overall, our findings provide imaging evidence for dissecting the heterogeneous mechanisms of cognitive impairment in anti-NMDAR encephalitis and support the further development of non-invasive, multimodal MRI biomarkers for disease monitoring and personalized management.

## Data Availability

The original contributions presented in the study are included in the article/supplementary material. Further inquiries can be directed to the corresponding author.
